# Bacterial Community Structure Dynamics in *Meloidogyne incognita*-Infected Roots and Its Role in Worm-Microbiome Interactions

**DOI:** 10.1128/mSphere.00306-20

**Published:** 2020-07-15

**Authors:** Timur M. Yergaliyev, Rivka Alexander-Shani, Hana Dimerets, Shimon Pivonia, David McK. Bird, Shimon Rachmilevitch, Amir Szitenberg

**Affiliations:** a Dead Sea and Arava Science Center, Dead Sea Branch, Masada, Israel; b The French Associates Institute for Agriculture and Biotechnology of Drylands, The Blaustein Institutes for Desert Research, Ben-Gurion University of the Negev, Beersheba, Israel; c Plant Protection Department, Agricultural Research and Development Station, Northern and Central Arava-Tamar, Sapir, Israel; d Department of Entomology and Plant Pathology, North Carolina State University, Raleigh, North Carolina, USA; University of Illinois at Urbana-Champaign

**Keywords:** *Meloidogyne incognita*, bacterial succession, microbiome, plant-microbe interactions, root knot nematode

## Abstract

Plant parasitic nematodes such as Meloidogyne incognita have a complex life cycle, occurring sequentially in various niches of the root and rhizosphere. They are known to form a range of interactions with bacteria and other microorganisms that can affect their densities and virulence. High-throughput sequencing can reveal these interactions in high temporal and geographic resolutions, although thus far we have only scratched the surface. In this study, we have carried out a longitudinal sampling scheme, repeatedly collecting rhizosphere soil, roots, galls, and second-stage juveniles from 20 plants to provide a high-resolution view of bacterial succession in these niches, using 16S rRNA metabarcoding. Our findings indicate that a structured community develops in the root, in which gall communities diverge from root segments lacking a gall, and that this structure is maintained throughout the crop season. We describe the successional process leading toward this structure, which is driven by interactions with the nematode and later by an increase in bacteria often found in hypoxic and anaerobic environments. We present evidence that this structure may play a role in the nematode’s chemotaxis toward uninfected root segments. Finally, we describe the J2 epibiotic microenvironment as ecologically deterministic, in part, due to the active bacterial attraction of second-stage juveniles.

**IMPORTANCE** The study of high-resolution successional processes within tightly linked microniches is rare. Using the power and relatively low cost of metabarcoding, we describe the bacterial succession and community structure in roots infected with root-knot nematodes and in the nematodes themselves. We reveal separate successional processes in galls and adjacent non-gall root sections, which are driven by the nematode’s life cycle and the progression of the crop season. With their relatively low genetic diversity, large geographic range, spatially complex life cycle, and the simplified agricultural ecosystems they occupy, root-knot nematodes can serve as a model organism for terrestrial holobiont ecology. This perspective can improve our understanding of the temporal and spatial aspects of biological control efficacy.

## Abstract (precis)

The study of high-resolution successional processes within tightly linked microniches is rare. Using the power and relatively low cost of metabarcoding, we describe the bacterial succession and community structure in roots infected with root-knot nematodes and in the nematodes themselves. We reveal separate successional processes in galls and adjacent non-gall root sections, which are driven by the nematode’s life cycle and the progression of the crop season. With their relatively low genetic diversity, large geographic range, spatially complex life cycle, and the simplified agricultural ecosystems they occupy, root-knot nematodes can serve as a model organism for terrestrial holobiont ecology. This perspective can improve our understanding of the temporal and spatial aspects of biological control efficacy.

## INTRODUCTION

Root-knot nematodes (RKN; genus *Meloidogyne*) are among the world’s most devastating plant pathogens, causing substantial yield losses in nearly all major agricultural crops (1). Meloidogyne incognita and closely related species are found in all regions that have mild winter temperatures (2), and they are regarded as one of the most serious threats to agriculture as climate change progresses (3). During their life cycle, *M. incognita* hatch in the soil and then invade a root. Thus, the nematodes are exposed to the soil microbiome, rhizobacteria, root epiphytes, and endophytes. Once inside the roots, the second-stage juveniles (J2) modify the cells in order to establish a feeding site and form the characteristic knots for which they are named. Each knot contains at least one nematode that feeds from a unique cell-type (the giant cells), surrounded by a gall of dividing cortical cells (4–7). Throughout their life cycle stages, *M. incognita* are known to interact with microbes, such as cellulase-secreting bacteria and plant effector-secreting bacteria, or bacterial and fungal antagonists (8–13). Consequently, it appears that the geographic or temporal variation in the bacterial and fungal communities of the rhizosphere and roots can partly explain the variable infestation success (8–11, 14–17) of such a near isogenic group of nematodes (18, 19).

The interaction between the virulence of *M. incognita* and the microbial diversity in the various niches *M. incognita* occupy has been studied in the context of biological control, revealing complex relationships, which efficacy diminishes with the transfer from lab to field (20). In this field of research, common themes include the isolation of *Meloidogyne* pathogens from the cuticles of J2s (21–23) and the identification of soil microbes and bacterial volatile compounds with antagonistic effects (24, 25) from key taxa, including *Rhizobia* (26), *Trichoderma* and *Pseudomonas* (27, 28), *Pasteuria* (29), *Pochonia* (30, 31), and some mycorrhiza (31–33).

Despite the importance of this plant parasite and its close ties with its cohabiting microbiome, microbial ecology studies seldom utilize deep-sequencing approaches. Very few studies have attempted to characterize the taxonomic and functional core microbiota (34, 35) or tie the microbial community composition in the soil or plant to RKN suppressiveness (36–39). In such studies, the temporal dynamics of the microbiome in each of the various niches occupied by nematodes at different life stages or throughout the crop season is rarely considered.

In this study, we sought to describe the rhizosphere, root, gall, and J2 bacterial succession across the primary nematode life cycle and throughout the crop season to understand how microbial interactions change with time. Temporal dynamics of the microbiome may affect the nematode’s life cycle and must be understood to successfully develop and apply biocontrol agents. To achieve this goal, we sampled the niches RKN occupy, at six time points, in 20 eggplant plants in southern Israel.

## RESULTS

To study the temporal dynamics of the bacterial community in the rhizosphere, roots, galls, and J2s in infected eggplant plants, we sampled these four niches from 20 plants at six time points throughout the crop season, which lasted 5 months. For each sample, we sequenced a 16S rRNA metabarcoding library, based on the V3-V4 hypervariable regions, on the Illumina MiSeq platform yielding 34,073,619 sequence read pairs. A curated data set of 306 samples, containing 10,416 amplicon sequence variants (ASVs), was retained following sequence error correction, chimera detection, and the exclusion of organelle sequences, low-abundance variants, and low-abundance samples (see Materials and Methods). This data set included 150 infected rhizosphere samples, 74 root samples, 61 gall samples, and 21 J2 samples. To describe the succession of bacteria throughout the crop season, we always selected the largest galls in the root sample and an adjacent infected root fragment lacking a gall (here referred to as “infected root”). Among the retained samples, read counts ranged between 8,828 and 107,687, with an average read count of 30,145. According to alpha rarefaction curves (see Fig. S1 at https://doi.org/10.6084/m9.figshare.12349328.v1) a sequencing depth of 8,828 was sufficient to capture rare taxa. The bioinformatics analysis carried out for this paper is available as Jupyter notebooks, along with input and output files, in a GitHub repository (40) and on Zenodo (https://doi.org/10.5281/zenodo.3731868) (41).

### Taxonomic bacterial community composition of the rhizosphere soil, root, gall, and J2 samples.

**FIG 1:**
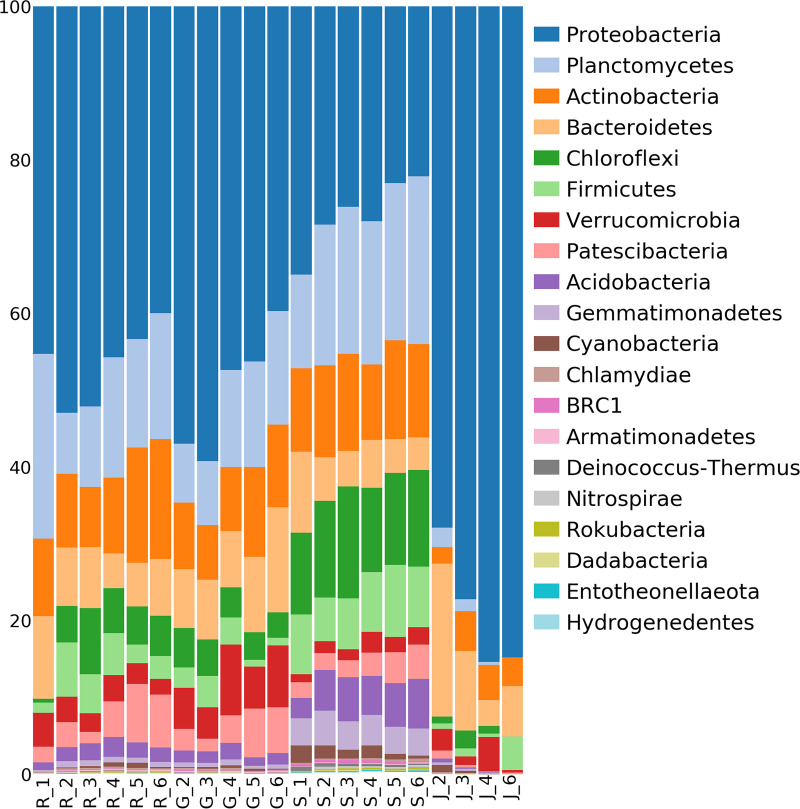
Relative abundances of bacteria. Phylum-level community compositions (20 most abundant phyla), pooled by niche and time point. R, root section lacking a gall; G, gall; S, rhizosphere soil; J, second-stage juvenile (J2). The integers represent time points 1 to 6.

Bacteria in the system belonged to 37 phyla and varied greatly among niches (Fig. 1). The most abundant phylum was *Proteobacteria*, followed by *Planctomycetes*, *Actinobacteria*, and *Chloroflexi*. In rhizosphere soil samples, averaging across all time points, 36 phyla were detected, the most abundant being *Proteobacteria* (27.3%), *Planctomycetes* (18.3%), *Chloroflexi* (11.7%), *Actinobacteria* (11.4%), *Firmicutes* (7.7%), and *Bacteroidetes* (6.2%). Of all niches, the relative abundance of *Proteobacteria* was lowest in the rhizosphere soil. Microbiomes of infected root and gall samples contained 33 and 34 phyla, respectively, and were very similar to each other on the phylum level. In both infected root and gall samples, the most abundant phyla were *Proteobacteria* (42.8 and 44.5%), *Planctomycetes* (15.9 and 13.4%), *Actinobacteria* (12.5 and 10.2%), and *Bacteroidetes* (7.3 and 10.6%). However, differences in *Firmicutes* (3.4 and 1.9%; Mann-Whitney test *q* value = 0.02) and *Verrucomicrobia* (2.9 and 6.5%; *q* value < 0.0001) were observed between the two niches. *Proteobacteria* was the most abundant phylum in DNA samples from J2s as well (74.8%), almost dominating the community. However, in contrast with the rhizosphere soil, in infected-root and gall samples, *Bacteroidetes* (12.7%) was relatively more abundant than *Actinobacteria* (0.3%; *q* value < 0.0001) and *Planctomycetes* (1.8%; *q* value < 0.0001). *Verrucomicrobia* (1.7%), *Firmicutes* (1.6%), *Chloroflexi* (1.4%), *Patescibacteria* (0.7%), *Cyanobacteria* (0.5%), and *Acidobacteria* (0.4%) were also observed in J2s.

Although *Proteobacteria* was the most abundant phylum in all the sample types, the subphylum *Gammaproteobacteria* dominated the *Proteobacteria* community in J2 DNA samples (55.4%), whereas *Alphaproteobacteria* represented the *Proteobacteria* in the other niches. The relative abundances of *Proteobacteria* decreased in all niches as time progressed, except in J2s, where they increased with time. In contrast, *Planctomycetes* were the most abundant phylum in the rhizosphere soil (17 to 22%) and their relative abundance increased with time. Root samples taken prior to planting, demonstrated notably higher relative abundances of *Planctomycetes*, which decreased after planting. In J2s, unlike other sample types, the relative abundances of *Planctomycetes* decreased with time.

### Alpha diversity.

**FIG 2:**
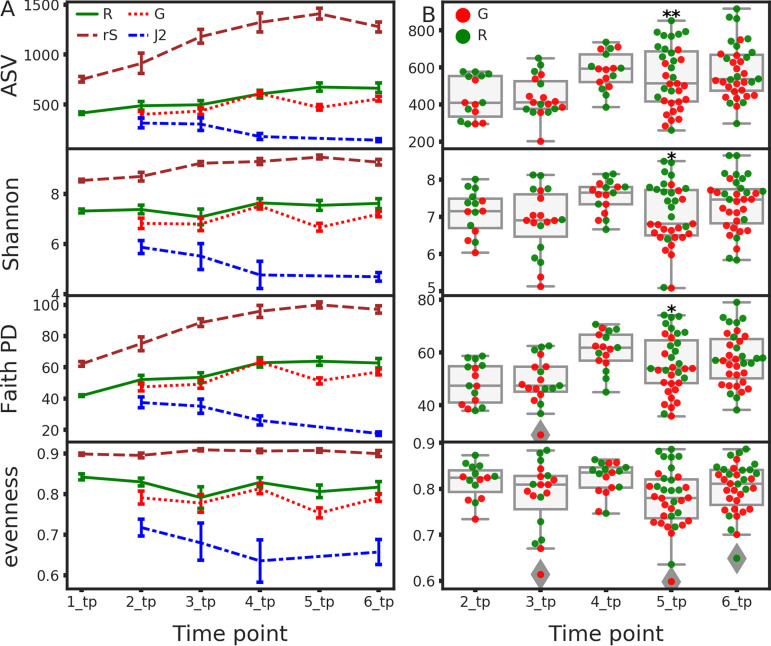
Alpha-diversity indices. (A) Longitudinal representation of alpha diversity indices (Shannon’s diversity index, Faith’s PD, observed ASVs, and evenness) for each niche (R, roots; G, galls; rS, rhizosphere soil; J2, second-stage juveniles). The error bars represent the standard deviations. (B) Alpha-diversity index distributions in gall (G) and infected root (R) samples from time points 2 to 6. Significant differences between galls and roots, according to a Kruskal-Wallis test, are indicated (*, *q* value < 0.05; **, *q* value < 0.01).

To study the temporal changes in alpha diversity during the growing season, we calculated the total observed ASV, Pielou’s evenness (42), Shannon’s diversity (43), and Faith’s phylogenetic diversity (Faith’s PD) (44) indices in each sample. We then summarized them by niche at each time point (Fig. 2A). ASV count increased moderately throughout the crop season in the infected root samples (Fig. 2A, solid green line) and more drastically in the rhizosphere (dashed brown line). This increase did not affect the phylogenetic diversity of ASVs, since it was accompanied by a similar increase in Faith’s PD, whereas the Shannon’s and Pielou’s indices were only very slightly perturbed. The temporal increase of alpha diversity in the roots as time progressed corresponded with the increase in alpha diversity in the rhizosphere.

The alpha diversity in gall samples diverged from that of infected roots toward the end of the crop season. Considering all the metrics, which have decreased in time point 5 in comparison with the infected root samples (Fig. 2B, Kruskal-Wallis *q* value = 0.008, 0.021, and 0.011 for the observed ASVs, Shannon’s diversity index, and Faith’s PD, respectively), this was likely due to an increase in relative abundance of a previously existing and phylogenetically narrow cohort of ASVs in the galls. J2s, collected from root surfaces starting at time point 2, had lower alpha diversity measures than other sample types, and they decreased further throughout the crop season. The decrease was evident in all indices, indicating that a phylogenetically narrow group of ASVs gradually took over the J2 cuticle community as time progressed.

### Beta diversity.

Beta diversity analyses were performed in order to study temporal and niche effects on the composition of the bacterial community. Weighted and unweighted pairwise UniFrac distances (45) were computed to account for changes in relative abundances or in the presence or absence of ASVs, respectively. Principal-coordinate analysis (PCoA) (46, 47) and biplots (47) were then used to visualize the relationships among the different data classes, and the key ASVs that explain them. ASVs were referred to by both their taxonomic assignment and the first six digits of their MD5 digests, referring to the full digests, as they appear in the representative sequences fasta file and biom table.

**FIG 3:**
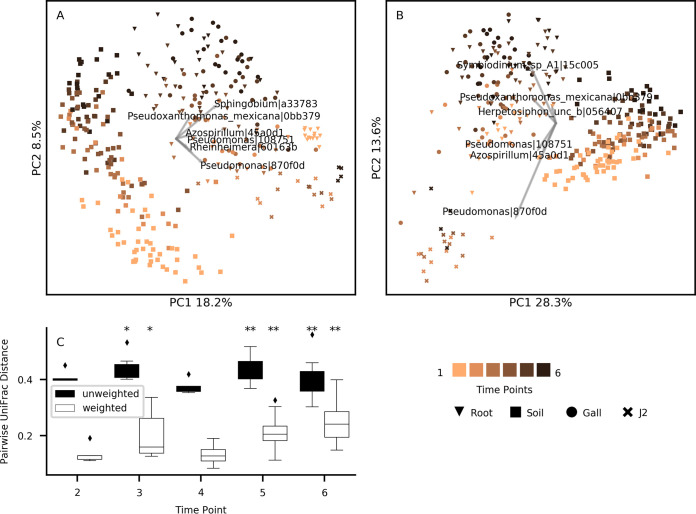
UniFrac distances among samples. (A to C) Unweighted (A) and weighted (B) UniFrac PCoA ordinations and biplots, as well as the distribution of pairwise UniFrac distances between infected root and gall samples at time points 2 to 6 (C). The markers represent the different niches and time points. Explanatory ASVs are denoted by the genus they were assigned to and the first six characters of their MD5 digest. Asterisks denote significant Wilcoxon test results (*, *q* value < 0.05; **, *q* value < 0.01).

In the PCoA analysis, unweighted (Fig. 3A) and weighted (Fig. 3B) UniFrac distances among the samples were explained by the niche of origin (axis 1, explaining 18.2 and 28.3% of the total variance, for unweighted and weighted UniFrac distances) and by time (axis 2, explaining 8.5 and 13.6% of the total variance). J2 samples were least affected by time and were most similar to early season roots throughout the season, mostly in terms of ASV presence and absence (Fig. 3A). Nevertheless, they were distinctly different from the root sample communities, suggesting that the composition of the nematode-associated bacterial community is quite unique and notably different from other niches. Biplot results revealed an increase in the relative abundance of ASVs assigned to *Pseudomonas* in J2s from all time points, and early season roots, compared to other sample classes (Fig. 3B), in line with the results of Harkes et al. (48), as well as the increase in ASVs assigned to *Azospirillum* and *Reinheimera*.

Gall samples (sphere markers; Fig. 3) differentiated from root (triangle markers; Fig. 3) communities in late season time points, but with some overlap between the two niches. Pairwise Wilcoxon paired tests (49) and permutational multivariate analysis of variance (PERMANOVA) tests (50) were conducted to test whether the observed differentiation of infected root and gall samples as the season progressed was significant. For time points 5 and 6, the distance between the sample types was significantly greater than zero (*q* value < 0.01), for both tests and both distance measures, but more discernible for the weighted UniFrac distances (Fig. 3C). When tested using PERMANOVA, the weighted UniFrac distance between root and galls samples was significantly greater than zero at time point 4 as well (*q* value = 0.024). The stronger signal received from the weighted UniFrac distance indicates that this divergence is primarily due to the few bacteria which took over the gall communities, and secondarily due to the introduction of new bacteria.

**FIG 4:**
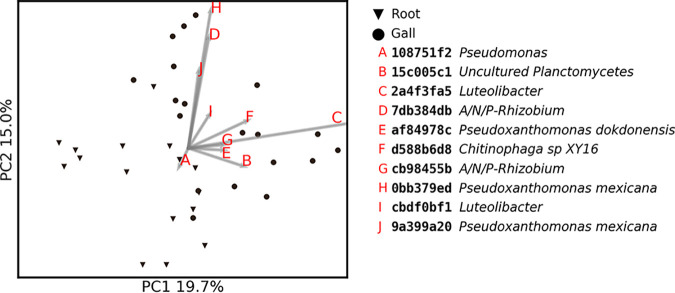
Weighted UniFrac PCoA and biplot for infected root and gall samples from time point 6. Marker shapes represent the niches. The 10 most important explanatory ASVs are represented by the first eight characters of their MD5 digest and their lowest identified taxonomic level.

To identify the ASVs responsible for the differentiation between gall and infected root samples, we repeated the PCoA and Biplot analyses, including only galls and infected root samples from the last time point (Fig. 4). Using the weighted UniFrac distance matrix, the combination of axes 1 and 2 segregated the two niches almost completely with 19.7 and 15.0% of the variance explained. Key ASVs responsible for this segregation belonged to *Planctomycetes*, *Pseudoxanthomonas* spp., genera from the *Rhizobium* clade (*Allorhizobium*, *Neorhizobium*, *Pararhizobium*, and *Rhizobium*, *sensu* Mousavi et al. [51], here referred to as “A/N/P-*Rhizobium*”), *Lechevalieria*, *Luteolibacter*, *Pseudomonas*, and *Saccharimonadales*. Only *Pseudomonas* were relatively more abundant in the root samples, while the rest of the ASVs were relatively more abundant in the gall samples.

### Cooccurrence of congeneric core ASVs and their phylogenetic relationships.

We treated the ratio between the core ASVs and the core taxa of each niche as a relative ecological-drift measure. A core genus without a respective core ASV is represented by different ASVs in different samples of the niche, indicating that the ecological constraints favoring one species or strain over another are not strong. The larger the deficit in core ASVs compared to the core taxa they are assigned to, the lower the ratio and the larger the drift. We based this approach on the notion that ecological drift can increase the genetic diversity among samples that were obtained from one niche (52). We formulated the difference as the ratio of core ASV count to core taxa count (ASV/taxon ratio [*R*]).

**FIG 5:**
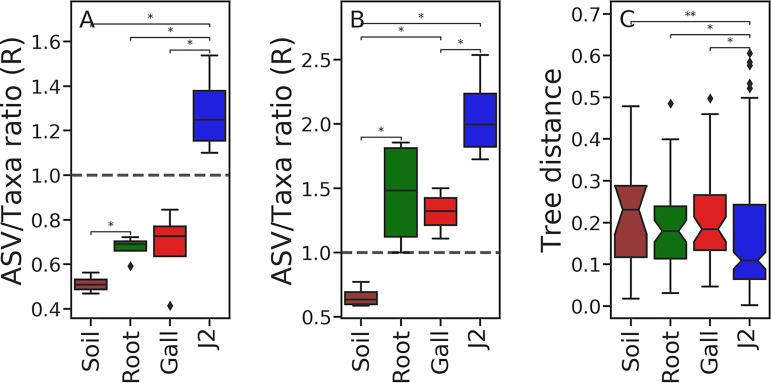
Comparative ecological drift in the sampled niches. The ratio (*R*) between all core ASV counts and all core taxa counts (A) or between the unique core ASV count and unique core taxa count (B) as a relative measure of ecological drift in each niche. (C) The distribution of pairwise patristic distances between congeneric ASVs was also used to compare stochasticity among the niches. Only time points 2, 3, 4, and 6 were used in all analyses.

With this notion in mind, among all the niches, only J2 had *R *>* *1, on average, in their 100% core microbiome (Fig. 5A). The *R* value in J2, was significantly different from *R* in other niches (*q* value < 0.023), in which the ASV count was lower than the taxa count. In addition, *R* in the infected root samples was also significantly greater than *R* in the rhizosphere soil (*q* value = 0.023). When considering only the unique taxa in each niche (Fig. 5B), galls and infected roots also had *R *>* *1, significantly greater than the *R* value of the rhizosphere soil (*q* value < 0.023). Consequently, J2s appear to provide a more selective microenvironment than the other niches, but the root and gall microenvironments have an intermediate level of stochasticity, between the rhizosphere soil and J2 niches.

In addition, we compared the strength of the deterministic forces shaping the communities in the various niches using a phylogenetic approach. For each niche, we computed the pairwise patristic distances between core ASVs sharing a taxon assignment. We expected a very selective niche to sustain congeneric ASVs that are more closely related to one another than congeneric ASVs in a more stochastic niche (Fig. 5C). J2s displayed a significantly lower median partistic distance than other niches (*q* value < 0.014), indicating that congeneric ASVs in the J2 samples are more closely related than congeneric ASVs in other niches.

### Bacterial succession.

**FIG 6:**
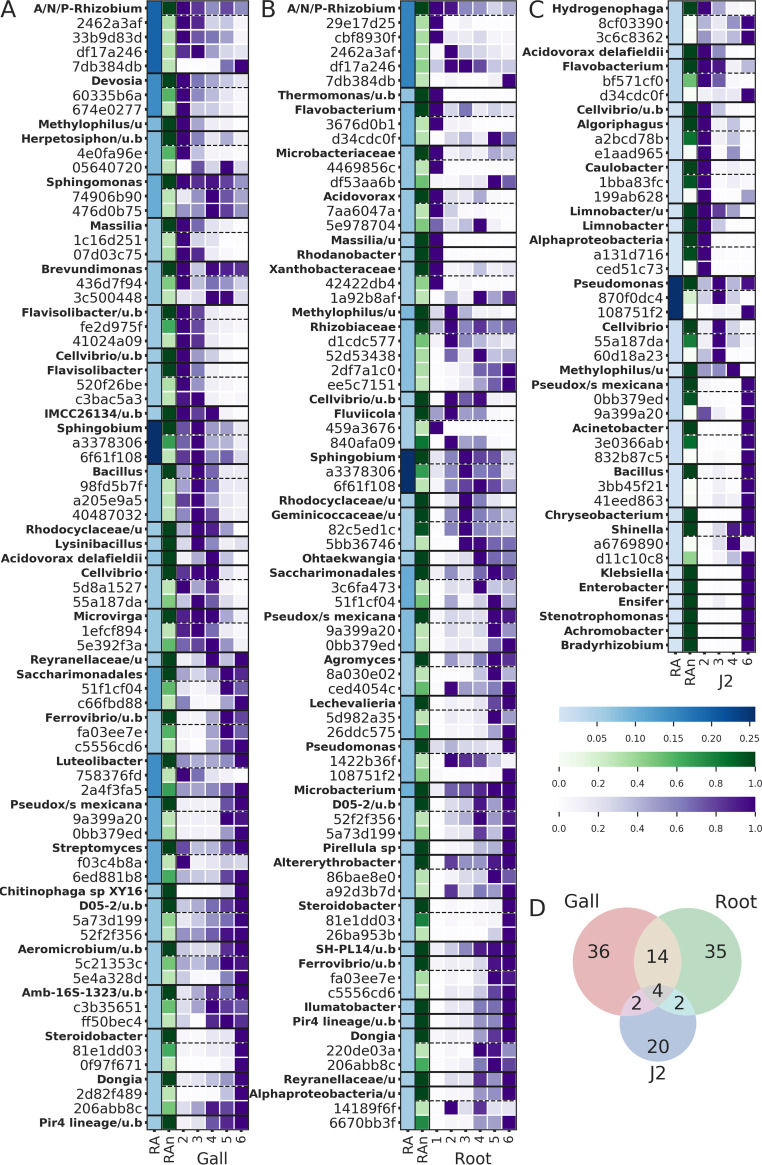
Bacterial succession of dynamic and important features. The most “important” and “dynamic” features were detected using a feature volatility analysis (see Materials and Methods). The curated cohort was selected separately for the galls (A), infected roots (B), and J2s (C) and includes taxa (indicated in boldface) and the most “dynamic” or “important” ASVs assigned to those taxa. The blue color scale denotes the average relative abundance (*RA*) of each taxon (boldface labels only) across all time points. The green color scale denotes the proportion of each ASV out of all the reads assigned to a given taxon, which we termed “relative abundance, normalized” (*RAn*). Thus, *RAn *=* *1 for the taxa themselves (in boldface). *RAn* can be quantitatively compared among ASVs assigned to the same taxon. The purple color scale represents the temporal distribution of each feature (the taxa or any of their ASVs), normalized by the peak relative abundance of the feature. Each heat map is sorted according to the peaking time point of the taxa (boldface), with taxa peaking at time point 1 or 2 at the top and taxa peaking at time point 6 at the bottom. The ASVs, however, appear immediately following the taxa they are assigned to, regardless of their peaking time point. (D) Number of shared and unique ASVs in the curated list of each niche indicated in a Venn diagram.

In addition to the temporal dynamics of alpha and beta diversity, we investigated the temporal change of discrete features (ASVs or taxa) to characterize the bacterial succession in various niches. We focused our investigation on features that we identified as “important” or “dynamic” (see Materials and Methods), based on an analysis of feature volatility (53). We also investigated the two most abundant ASVs belonging to the included taxa, where they were not already considered. The mean relative abundance of features is presented as a heatmap (Fig. 6), organized according to niche (Fig. 6A to C) and time point. Blue shades represent the relative abundance of each taxon across the time points, green shades represent the relative abundance of each ASV within the pool of ASVs assigned to a certain taxon, and the purple shades represent the temporal distribution of the ASV or taxon. Dashed borders separate a taxon and the ASVs assigned to it.

A comparison of the three heatmaps reveals largely independent sets of ASVs in each niche, which most explain temporal changes, as illustrated by the Venn diagram (Fig. 6D). Within the galls (Fig. 6A), an “early” bacterial community, including 14 taxa (A/N/P-*Rhizobium*, *Devosia*, *Methylophilus*, *Herpetosiphon*, *Sphingomonas*, *Massilia*, *Brevundimonas*, *Flavisolibacter*, *Cellvibrio*, the uncultured *Verrucomicrobia* IMCC26134, *Sphingobium*, *Bacillus*, *Rhodocyclaceae*, Acidovorax delafieldii, and *Microvirga*), is gradually displaced by a “late” gall community, including 12 taxa (*Reyranellaceae*, *Saccharimonadales*, *Ferrovibrio*, *Luteolibacter*, Pseudoxanthomonas mexicana, *Streptomyces*, *Chitinophaga*, *Aeromicrobium*, the uncultured *Rhizobiales* Amb-16S-1323, *Steroidobacter*, *Dongia*, and uncultured *Planctomycetaceae* sp. belonging to lineage Pir4). This displacement already begins and intensifies within the primary nematode life cycle in time points 2 and 3, earlier than a similar process that occurs in the root, for many taxa.

Another important aspect of the gall bacterial succession is the origin of taxa. Only a few taxa in the root clearly originated from the naive roots (the root community prior to planting). In the early community, these include *Methylophilus*, *Sphingobium*, and Acidovorax delafieldii (see Fig. S2 at https://doi.org/10.6084/m9.figshare.12349337.v1). Of these, only *Sphingobium* persisted successfully throughout most of the crop season. Conversely, most of the genera detected in the galls emerged from the rhizosphere soil, and were sometimes displaced by congenerics at later time points. Most notably, A/N/P*-Rhizobium* were already present in the naive roots, but then were effectively outcompeted by rhizosphere soil congenerics, which then shared the gall A/N/P-*Rhizobium* community (e.g., root originated ASV cbf5930fe versus rhizosphere soil originated ASV 33b9d83d1; see Fig. S2 at https://doi.org/10.6084/m9.figshare.12349337.v1). In the late season gall community, only *Luteolibacter* clearly emerged from the naive roots and not from the rhizosphere soil (Fig. S2)*. Luteolibacter* represented ∼7% of the gall community by the end of the season. ASV 2a4f3fa50 outcompeted other congeneric ASVs to monopolize the *Luteolibacter* community.

### Attraction assay.

**FIG 7:**
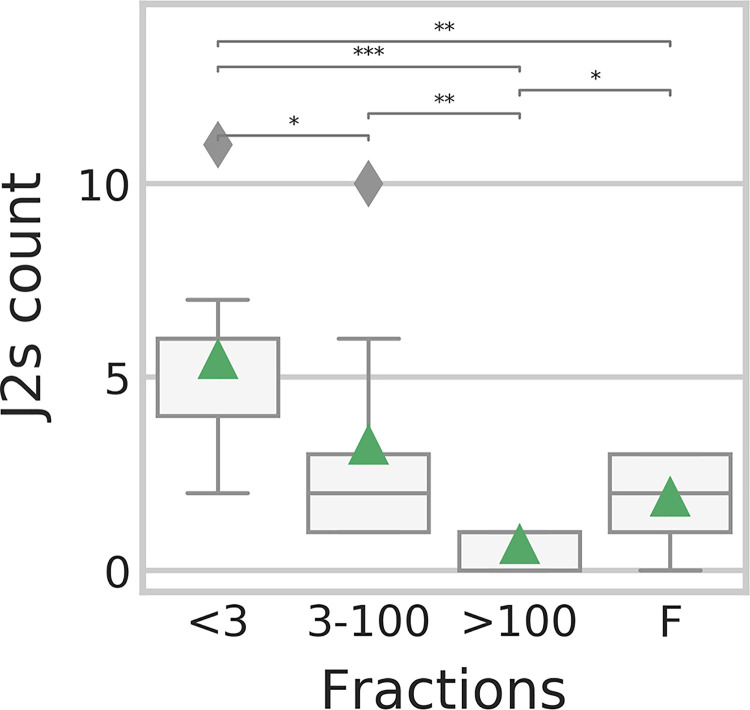
Attraction assay. The number of J2 nematodes attracted to a *Pseudomonas* filtrate, when presented with both the filtrate and a root fragment. *x* axis, size of compounds in the filtrate (kDa); F, whole filtrate; *y* axis, J2 nematode count. Asterisks indicate significant differences according to Mann-Whitney U test (***, *q* value < 0.001; **, *q* value < 0.01; *, *q* value < 0.05).

In 2017, gall bearing eggplant roots were sliced and washed with PBS in an attempt to isolate RKN related bacteria (see File S1 at https://doi.org/10.6084/m9.figshare.12349349.v1). Since RKN pathogens often actively attract J2s, we carried out an attraction assay, testing the attraction of J2s to each of two isolates, given the isolate and fresh root as options, or the sterile medium and a fresh root as control. The attraction of J2s to one isolate in particular was 10-fold higher than to the root. Sanger sequencing of the isolate (File S1) revealed that its V3-V4 region sequence was identical to *Pseudomonas* 108751f2 (Fig. 4). To test whether compounds secreted by this isolate can attract J2s, we carried out an additional attraction assay, in which J2s were allowed to select between a fresh root fragment and one of the following: whole bacterial medium filtrate, containing the isolate’s exudates; the <3-kDa fraction of this filtrate; the 3- to 100-kDa fraction of the filtrate; and the fraction of compounds >100 kDa (see Materials and Methods). The results, summarized in Fig. 7, show that J2s are significantly more attracted to the <3-kDa fraction of the filtrate than to the other fractions or the whole filtrate (0.0009 < *q* value < 0.027).

## DISCUSSION

### Root community structure at the end of the crop season.

Animals and plants host a large number of interacting microbes, which can modulate the functions and behaviors within the complex. This microbial community, like any other ecological community, is subject to succession. In this study, we investigated the structure of the bacterial community in RKN infected roots and its interaction with the communities in the rhizosphere soil and J2 nematodes, with respect to the community dynamics along the nematode’s primary life cycle and the crop season. According to beta-diversity indices (Fig. 4), gall communities diverge from those of adjacent root sections lacking a gall, late in the crop season, revealing a highly structured root community. According to alpha-diversity (Fig. 2B) and beta-diversity (Fig. 3C) pairwise tests, this structure starts to develop even earlier in the season.

The mature gall community differs from that of adjacent root segments by the higher relative abundance of bacteria with known nematostatic (*Pseudoxanthomonas* spp. [54]) and antibiotic (*Lechevalieria* sp. [55–58]) activity, RKN symbionts participating in the structural modification of the root (A/N/P-*Rhizobium* [13]), nematode egg-shell feeding bacteria (*Chitinophaga* [59, 60]), or bacteria providing protection from reactive oxygen species (ROS) (*Luteolibacter* [61]). Conversely, the adjacent root segments present a higher relative abundance of a *Pseudomonas* sp. (ASV 108751f2), which increase occurred late in the season (Fig. 6B). This *Pseudomonas* sp. was isolated and experimentally proven to be highly attractive to J2, along with the exudate fraction, which contains molecules smaller than 3 kDa. It is thus possible that the structured bacterial community we have identified plays a role in providing soil J2s with the chemical cues they require to identify the healthier sections of the already deteriorated root system. In addition, some endophytic *Pseudomonas* spp. have been shown to degrade ROS and can thus further facilitate the repeated infection of parasitic nematodes (62, 63). The same *Pseudomonas* sp. strain (ASV 108751f2) was also detected on J2s in increasing densities at the end of the crop season (Fig. 6C). This could be an artifact of the Baermann tray method, but the evidence supporting J2’s active attraction to this isolate could point to a more elaborate form of interaction in which the bacterium first attracts the nematode to hitchhike into the root and, once established, can help guide the nematode into less abstracted sections of the root. Evidence of bacterial J2 hitchhikers, which perform some function inside the root, has been presented in the past (64). However, these bacteria were RKN pathogens that also elicited ROS production, a plant defense mechanism. Such an alternative mechanism may also explain our observations. A/N/P-*Rhizobium*, a taxon which occurred in the root, gall, and J2 samples, showed a persistent relative abundance in the gall and J2 samples, while its relative abundance decreased in the root segments (see Fig. S2 at https://doi.org/10.6084/m9.figshare.12349337.v1). In the past, it has been shown that bacteria belonging to this group were able to successfully invade plant hosts by using nematodes as a transition vector (65). Therefore, the observed covarying pattern between the roots and galls may be nonincidental.

### Bacterial succession in the gall along the primary RKN life cycle and the crop season.

Upon consideration of the most dynamic fractions of the bacterial communities, the processes leading to the result described above are exposed as a gradual community shift (Fig. 6A). The early gall community comprises bacteria which confer structural modifications to the root (A/N/P-*Rhizobium* [13], *Devosia* sp. [66], and then *Microvirga* [67]), fixing nitrogen (A/N/P-*Rhizobium* [68], *Devosia* sp. [66, 69, 70], *Microvirga* [67], *Sphingomonas* [71], and later *Cellvibrio* [72], *Microvirga* [67]. and possibly *Bacillus* [73]) and bacteria capable of degrading polysaccharides (*Herpetosiphon* [74, 75], *Massilia* [76], *Verrucomicrobia* [77, 78], *Cellvibrio* [79], *Sphingobium* [80], and possibly *Bacillus* [81]). While some potential chitin feeders form a part of this community early on (*Herpetosiphon* [82, 83], *Sphingomonas* [84], and *Massilia* [76]), time point 3, 40 days from planting and onward, sees a gradual addition of chitin feeders (*Cellvibrio* [85], *Lysinibacillus* [86], *Streptomyces* [87], and possibly *Bacillus* [86, 88]), possibly because of an increasing eggshell density.

Since we sampled the largest galls at each time point, the late season galls (time points 4 to 6) often represent already inactive galls. The most striking characteristic of the late season gall community is a rapid increase in bacteria capable of anaerobic or hypoxic growth. Only ASVs of *Sphingomonas*, known to include facultative anaerobic species (89), persisted throughout the season. Even a *Rhizobiales* representative present in the late season gall community (Amb-16S-1323) seems to occur in hypoxic and anaerobic environments, unlike the early season representatives of the order which are aerobic, plant-related bacteria (see Fig. S3 at https://doi.org/10.6084/m9.figshare.12349346.v1). Eight other genera, which ASVs increased during this time frame, have known anaerobic or hypoxic species (*Bacillus* [90, 91], *Rhodocyclaceae* [92], Acidovorax delafieldii [93], *Ferrovibrio* [94], Pseudoxanthomonas mexicana [95], *Chitinophaga* [59], *Steroidobacter* [96], and a Pir4 lineage bacterium [97]). Lastly, some of the taxa comprising the late season gall community have been associated with plant parasitic nematode soil suppressiveness or RKN antagonism (*Chitinophaga* and *Streptomyces* [98–102]).

### Second-stage juvenile epibiotic microbiome.

Previous studies (103–109) have shown that almost all bacteria that were found to be most important or dynamic in J2s (Fig. 6C) are associated with RKN control, although *Cellvibrio* may assist their root penetration (110). While the bacterial communities of the rhizosphere soil and root samples consistently shifted in time, according to UniFrac pairwise distances (Fig. 3A and B), the J2 community beta-diversity indices remained relatively very constant. Furthermore, most of the core genera in the J2 community were represented by the same ASVs in all samples and time points, unlike other niches, and cooccurring congeneric ASVs were more closely related in J2 samples than in other niches (Fig. 5). Presumably, the J2 cuticle is a very selective environment and specialist bacteria can predictably outcompete other bacteria that exist in the root or rhizosphere soil. It is possible that like many other organisms in which skin epibionts play a role in preventing pathogens from settling (111), these J2 epibionts may have to evade antimicrobial mechanisms exerted by the host. Concomitantly, some bacteria (14, 112) actively attract or attach to J2 nematodes and colonize their surfaces in various forms of symbiosis. The *Pseudomonas* sp. isolate, whose density increased in the J2 samples at the end of the crop season (Fig. 6C) and was shown to attract the J2 nematodes (Fig. 7), would also belong to this group of J2 symbionts. This is another mechanism maintaining the low genetic diversity of the J2 epibionts, which even reduces alpha diversity with time (Fig. 2A). Although the Baermann tray extraction method would potentially bias the result by contaminating the perceived J2 bacterial community with root and rhizosphere soil bacteria, we believe this effect was small, given the fact that the J2 microbiome remained constant, in spite of the very dynamic community in the other niches.

### Conclusion.

In this study, by using a longitudinal approach and a large number of replicates, we were able to robustly describe a bacterial community structure within *M. incognita*-infected roots. This structure seems to persevere until the end of the crop season and may play a role in the life cycle of the nematodes. During the crop season, bacteria found in hypoxic and anaerobic environments often increase; however, a connection to the nematode’s life cycle was also observed, particularly in connection to root structure modifications, polysaccharide metabolism, and chitin metabolism. With their large geographic range (2, 113), the relatively simplified agricultural ecosystems they occupy and their minimal genetic diversity (18, 19), *M. incognita* could be used as a much-needed model organism for terrestrial holobiont ecology studies. Understanding the effect of variations in the rhizosphere soil and host-crop microbial seed bank on the root community structure and its interactions with environmental factors may improve our understanding of ecological processes in terrestrial holobionts and the efficacy of biocontrol agents in field conditions (114).

## MATERIALS AND METHODS

### Sampling.

Samples were collected from greenhouse cultivated eggplant plants, infected by *M. incognita*, in Hatzeva (Israel). Twenty plants were repeatedly sampled throughout the crop season. At time point 1, root sections were collected prior to planting to represent the preexisting root endophytic community. At each subsequent time point (see Fig. S4 at https://doi.org/10.6084/m9.figshare.12408695.v1), we collected an entire lateral root branch from each plant, leaving the plant intact for future sampling. In the lab, we collected the rhizosphere soil and identified the largest galls available at the time. We then dissected the gall fragments and additional root segments lacking a gall, which were adjacent to the collected galls. The root segments were up to 3 cm long. In addition, roots were collected from each plant, in order to extract J2s, using the Baermann tray method, following Williamson and Čepulytė (115). The extracted J2s were filtered onto 47-mm-diameter and 0.45-μm-pore-size cellulose ester filters (GE Healthcare Whatman). All samples were kept at –80°C until DNA extraction. Table 1 summarizes the number of samples available for analysis after sequencing and data filtration (see below). Rhizosphere soil samples were also collected from additional plants and thus the number of soil samples exceeded 20 at most time points. The number of J2 samples in Table 1 was affected mostly by the density of J2s in the samples but also by subsequent data curation (see below).

**TABLE 1:** Sample collection

Time point	Date	No. of samples			
		Soil	Root	Gall	J2
1	20.12.17	44	14^*a*^		
2	14.01.18	16	9	6	8
3	30.01.18	21	9	10	7
4	25.02.18	26	9	8	2
5	26.03.18	24	17	18	
6	22.05.18	19	16	19	4

a Uninfected roots collected prior to planting.

### DNA extraction and 16S rRNA library preparation.

The roots were washed with 1% sodium hypochlorite solution and rinsed with distilled water, and then gall and adjacent root fragments were dissected. Each segment was cut into small pieces and placed in a well within a 96-well sample-plate. For rhizosphere soil samples, a 0.25-g sample was placed in each sample plate well. The DNA of root, gall, and soil was extracted by using a DNeasy PowerSoil kit (Qiagen) according to the manufacturer’s instructions. J2 DNA was extracted from the filters using a PowerWater DNA extraction kit (Qiagen), following the manufacturer’s instructions. Metabarcoding libraries were prepared as previously described (116), using a two-step PCR protocol. For the first PCR, the V3-V4 16S rRNA region (117) was amplified using the forward primer 5′-tcgtcggcagcgtcagatgtgtataagagacagCCTACGGGNGGCWGCAG-3′ and the reverse primer 5′-gtctcgtgggctcggagatgtgtataagagacagGACTACHVGGGTATCTAATCC-3′, along with artificial overhang sequences (lowercase). In the second PCR, sample-specific barcode sequences and Illumina flow cell adapters were attached using the forward primer 5′-AATGATACGGCGACCACCGAGATCTACACtcgtcggcagcgtcagatgtgtataagagacag-3′ and the reverse primer 5′-CAAGCAGAAGACGGCATACGAGATXXXXXXgtctcgtgggctcgg-3′, including Illumina adapters (uppercase), overhang complementary sequences (lowercase), and sample-specific DNA barcodes (“X” sequence). The PCRs were carried out in triplicate, with a KAPA HiFi HotStart ReadyMix PCR kit (KAPA Biosystems), in a volume of 25 μl, including 2 μl of DNA template and according to the manufacturer’s instructions. The first PCR started with a denaturation step of 3 min at 95°C, followed by 30 cycles of 20 s of denaturation at 98°C, 15 s of annealing at 55°C, and 7 s of polymerization at 72°C. The reaction was finalized with another 60-s polymerization step. The second PCR was carried out in a volume of 25 μl as well, with 2 μl of the PCR1 product as DNA template. Samples were first denatured for 3 min at 95°C and then underwent eight 20-s cycles of denaturation at 98°C, 15 s of annealing at 55°C, and 7 s of polymerization at 72°C. The second PCR was also finalized with another 60-s polymerization step. The first and second PCR products were purified using AMPure XP PCR product cleanup and size selection kit (Beckman Coulter), according to the manufacturer’s instructions, and sequenced on an Illumina MiSeq to produce 250-bp paired-end sequence reads. The sequencing was carried out by the Nancy and Stephen Grand Israel National Center for Personalized Medicine, The Weizmann Institute of Science.

### Bioinformatics.

**(i) Data processing, taxonomy assignment, and biodiversity analysis.** All analyses carried out for the present study are available as Jupyter notebooks in a GitHub repository (40, 41), along with the sequence data and intermediate and output files. The bioinformatics analysis was carried out with Qiime2 (v.2019.4/10) (118). Forward and reverse PCR primers were removed from the MiSeqs reads by using the q2-cutadapt plugin (119). Using the q2-DADA2 plugin (120), paired reads were truncated to 267 and 238 bp for the forward and reverse reads, respectively, and the first two bases were removed as well. The reads were then quality filtered, error corrected, dereplicated, and merged. Finally, chimeric sequences were removed, to produce the amplicon sequence variants (ASVs). For taxonomic assignment, a naive Bayes classifier was trained using taxonomy assigned reference sequences from the Silva SSU-rRNA database (v.132; 16S, 99%) (121). Reference sequences were trimmed to the V3-V4 fragment. All ASVs that were identified as mitochondrial or chloroplast sequences or that were assigned only to the bacteria level, as well as completely unassigned sequences, were filtered out from the feature table. The ASV biom formatted table was further filtered to exclude samples with fewer than 8,828 sequences. An ASV phylogenetic tree was built with the q2-phylogeny plugin, implementing MAFFT 7.3 (122) for sequence alignment, and FastTree 2.1 (123), with default masking options. Microbial diversity was estimated based on the number of ASVs observed, Pielou’s evenness (42), Shannon’s diversity indices (43), and Faith’s phylogenetic diversity (44) for alpha diversity and weighted and unweighted UniFrac distance (45) matrices for beta diversity. Following Glassman et al. (124), all indices were based on ASV data. Ordination of the beta-diversity distance was carried out with a principal-coordinate analysis (PCoA), and the key taxa explaining beta diversity was obtained using biplot analysis (46, 47). Tests for significant differences in alpha and beta diversity between groups of samples representing one niche and one time point were implemented with the Kruskal-Wallis test (alpha diversity) (125) or the Wilcoxon (49) and PERMANOVA (50) tests (beta diversity). We focused our attention on the gall and infected root sample types. *P* values were corrected for multiple testing using the Benjamini-Hochberg procedure (126). Corrected *P* values are referred to as “*q* values” throughout the text. In the Qiime2 environment, the MD5 message-digest algorithm is used to name ASVs by their sequence digest. To make these digests more human readable, we prefixed each digest with the lowest available taxonomic level and the “|” symbol. This change was consistently implemented in the biome table, the representative sequences fasta file, and the taxonomy assignment table.

**(ii) Longitudinal feature volatility analysis.** Qiime2 feature volatility longitudinal analysis (53) was carried out to study the temporal changes in relative abundances of each ASV and taxon, separately in the galls, roots. and J2. Both ASVs and taxa were analyzed, assuming that in some cases discrete ASVs that represent functionally and taxonomically similar bacteria are ecologically interchangeable. In such cases, patterns that would be observed at the species or genus level, might be lost at the ASV level, and vice versa. We grouped ASVs into taxa based on their lowest available taxonomic level. One parameter used to identify key ASVs and taxa in the system was “importance.” Importance is defined as the Euclidean distance of the relative abundance vector of a given taxon or ASV from a null vector of the same length (53). For each niche, we identified the 15 most important ASVs and 15 most important taxa. We additionally included the 10 ASVs and 10 taxa with the highest net average change among time points (five increasing and five decreasing). Lastly, we included the two most abundant ASVs of the included taxa, where they were not already represented. In addition, for each ASV on the list we added the taxon it was assigned to, where it was not already included. Figure 6 summarizes the results of this analysis as a heatmap. It additionally includes a Venn diagram of the ASVs that are represented in the heatmap.

**(iii) Core ASV/taxon ratios and tree distances.** To evaluate the relative strength of ecological drift among niches, we computed ASV/taxon count ratios (*R*) within the core microbiome and unique microbiome of each niche and time point. The core microbiome of a given niche at a given time point (e.g., galls at time point 2) consists of either ASVs that occur in all the samples of the group (core ASVs) or genera that occur in all the samples of the group (core taxa). The unique microbiome includes the subset of the core microbiome, which occurs only in that niche, at a given time point.

Because ecological drift is expected to increase the genetic diversity among samples (51), we relied on the underlying assumption that the ratio of core ASV count to core taxa count is associated with ecological drift. If a niche is very deterministic and only highly adapted bacteria can survive there, a given genus, which occurs in all the samples from that niche, will be represented by the same ASV(s) in all samples of that niche. Conversely, if a niche is not limiting at all in its conditions, strains or species belonging to this genus will exist in random proportions in each sample of that niche and the chance of any given ASV to be included in all of the samples from that niche will be low.

We therefore infer that if many genera are represented by the same set of ASVs in all the samples (i.e., core ASV/core taxon ratio of ≥1) then the conditions in this niche must be very limiting to genetic diversity (deterministic niche), but if many taxa are represented by different ASVs in each sample (i.e., core ASV/core taxon ratio of <<1), then this niche is not limiting to genetic diversity (stochastic niche). The core taxa count to core ASV count ratio is a relative measure and is therefore unitless.

Core ASV and core taxa were determined by the Qiime2 feature-table plugin separately for several subsets, each containing samples of one niche and one time point, either time point 2, 3, 4, or 6. Only ASVs or taxa that were detected in all the samples of a given subset were included (100% core microbiome). Another data set, containing only the core ASVs and taxa that were unique to each niche, was produced. *R* values were computed for each subset, for the core microbiome and the unique microbiome. The distribution of core and unique *R* values are presented in Fig. 5A and B, respectively. Pairwise comparisons between niches were tested with Mann-Whitney U tests (127), corrected for multiple testing using the Benjamini-Hochberg procedure (126).

Since we suspected that the *R* values might be influenced by differences in sample sizes among the subsets, we repeated the analysis with a normalized sample size. To normalize the sample size, we constrained the number of samples in each time point. To do that, we identified the niche with the lowest number of samples in each time point and reduced the number of samples in the remaining niches to conform with that number. The resulting *R* value distributions and the pairwise Mann-Whitney U tests were similar to those obtained with the full data set.

A genetic signature of congeneric ASV divergence would also be recorded in their pairwise phylogenetic distances. For each group of congeneric ASVs in the core microbiome, a phylogenetic tree was reconstructed, from which we obtained pairwise patristic distances of congeneric ASVs. Then, the distribution of congeneric ASV patristic distances was computed for each subset and presented in Fig. 5C. The phylogenetic trees were reconstructed as follows: for each genus level taxon, we produced a fasta file of ASVs. The 50 best matches in the Silva 138 16S rRNA database (121) were identified for each ASV, using blastn 2.9.0+ (128). The ASV and reference sequences were aligned using the L-ins-i algorithm implemented in MAFFT 7.3 (129) and trimmed by trimAl (130), with a 0.1 gap threshold. Maximum-likelihood trees were constructed with RAxML 8.2 (131) using the GTR-Gamma model of sequence evolution. Pairwise comparisons of patristic distance distributions were tested with Mann-Whitney U tests (127), corrected for multiple testing with the Benjamini-Hochberg procedure (126).

**(iv) Isolation sources of early and late gall community *Rhizobiales*.** The following steps were taken to summarize the isolation sources of three *Rhizobiales* taxa from the gall community, including the group of uncultured *Rhizobiales* Amb-16S-1323, *Devosia* spp., and the A/N/P-*Rhizobium* cluster. For each taxon, all the sequences available on SILVA (121) were downloaded as fasta files, and their GenBank entries were retrieved based on their accession numbers, using the BioPython Entrez python module (132). Isolation sources, taken from the isolation_source qualifier in the GenBank entries, were categorized into 18 categories, which were then summarized and are presented in Fig. S3 at https://doi.org/10.6084/m9.figshare.12349346.v1.

### Attraction assay.

Preliminary results (see File S1 at https://doi.org/10.6084/m9.figshare.12349349.v1) revealed that J2s were attracted to the volatiles of a *Pseudomonas* sp. isolate, which shared its V3V4 region sequence with ASV 108751f28645926db7b461f27b822162. To test the chemical attraction of J2s to the isolate’s volatiles, we carried out attraction assays, according to the method of Williamson and Čepulytė (115). The attraction assay was carried out in 12-well plates (Greiner Bio-One) containing Pluronic-127 gel, which is a transparent and porous medium. Each well contained both a 2-cm fragment of a fresh basil root and a 10-μl pipette tip containing an attractant to compare between the attraction to the liquid in the tip and the root (see Fig. 1 in File S1 at https://doi.org/10.6084/m9.figshare.12349349.v1). The tip contained either the whole bacterial filtrate (excluding the bacterium) or one of three size determined fractions (<3 kDa, 3 to 100 kDa, and >100 kDa), in nine replicates per treatment. Then, 1 ml of Pluronic-F127 Tris-MES buffer gel (Sigma-Aldrich) containing ∼50 J2s was added to each well. A dissection microscope was used to record the number of J2s in each tip after 24 h (Fig. 7).

The attractants we introduced in the pipette tips were prepared as follows: the isolate was incubated in aqueous beef-extract peptone medium (beef extract, 3 g liter^−1^; peptone, 10 g liter^−1^; NaCl, 5 g liter^−1^ [133]) for 48 h at 37°C. The culture was filtered using a 0.45-μm syringe filter to obtain the bacterial extracts (whole filtrate). The size fractions were then separated using Amicon Ultra-15 centrifugal filter units with Ultracel-PL membrane according to the manufacturer’s instructions. *M. incognita* eggs were sieved from infected tomato roots using a set of mesh #200 sieve on top of mesh #500 sieve (W. S. Tyler), and J2s were hatched with a Baermann tray, as described by Williamson and Čepulytė (115).

### Data availability.

The data sets generated and analyzed during the present study are available from the National Center for Biotechnology Information BioProject repository under accession number PRJNA614519. Data and script are archived as a GitHub release (40), Zenodo (41).
